# Combination of oral antibiotics and mechanical bowel preparation for colorectal surgery

**DOI:** 10.1002/ags3.12064

**Published:** 2018-03-25

**Authors:** Chu Matsuda, Hugh Colvin, Yosuke Adachi

**Affiliations:** ^1^ Department of Gastroenterological Surgery Graduate School of Medicine Osaka University Suita Japan; ^2^ Department of General Surgery National Health Service Fife Kirkcaldy UK; ^3^ Center for the Study of Medical Education School of Medicine Kurume University Kurume Japan

Traditionally, oral mechanical bowel preparation (OMBP) has been used preoperatively for patients undergoing elective colorectal surgery to reduce postoperative complications. Contrary to the traditional practice, several recent trials and meta‐analyses have failed to show any significant benefit of using OMBP in elective colorectal resections.^1^

However, the use of OMBP in elective colorectal surgery is supported by emerging evidence when it is used together with oral antibiotics.

Using National Surgical Quality Improvement Program (NSQIP)‐targeted colectomy data initiated in 2012, Kiran et al compared the impact of preoperative OMBP and oral antibiotics (OMBP+/ABX+), OMBP alone (OMBP+/ABX−), or no bowel preparation (No‐prep) on outcomes for patients undergoing elective colorectal resection. Of 8442 patients, 27.2% had No‐prep, 45.3% had OMBP+/ABX−, and 27.5% had OMBP+/ABX+. OMBP+/ABX+ was independently associated with reduced postoperative ileus (OR = 0.71, 95% CI: 0.56‐0.90), surgical site infection (SSI) (OR = 0.40, 95% CI: 0.31‐0.53) and anastomotic leak (OR = 0.57, 95% CI: 0.35‐0.94) when compared to No‐prep on multivariate analysis. OMBP+/ABX− did not reduce postoperative complications when compared to No‐prep, consistent with findings from other recent studies.^2^

The importance of oral antibiotics in addition to OMBP in elective colorectal surgery is emphasized further by Chen et al who analyzed seven randomized controlled trials that consisted of 1769 patients. Both total and incisional SSI rates were significantly reduced in patients who received oral and systemic antibiotics together with OMBP, compared with patients who received systemic antibiotics alone together with OMBP (total SSI: 7.2% vs 16.0%, *P* < .00001; incisional SSI: 4.6% vs 12.1%, *P* < .00001).^3^

The significance of OMBP and oral antibiotics over other measures in reducing postoperative infections is highlighted by Ohman et al in a prospective study. They investigated the use of oral antibiotics and OMBP in elective colorectal surgery, together with other measures as part of an infection prevention bundle (IPB) that included preoperative shower with chlorhexidine, hair removal and skin preparation with antibiotic wound irrigation before the wound closure. For the 24 months before IPB introduction, the overall SSI rate was 19.7%. During the 30 months after IPB implementation, the SSI rate decreased to 8.2% (*P* < .0001). On multivariate analysis, full bowel preparation was independently associated with significantly fewer SSI [OR 0.2, 95% CI 0.1‐0.9, *P* < .01]. The authors concluded that the combination of oral antibiotics with mechanical bowel preparation was the strongest predictor of decreased SSI.^4^

There is ongoing debate on the role of mechanical bowel preparation and oral antibiotics in elective colorectal surgery. The recent studies show that OMBP together with oral antibiotics improves outcomes after colorectal resection.
